# Riboseq-flow: A streamlined, reliable pipeline for ribosome profiling data analysis and quality control

**DOI:** 10.12688/wellcomeopenres.21000.1

**Published:** 2024-04-11

**Authors:** Ira A. Iosub, Oscar G. Wilkins, Jernej Ule

**Affiliations:** 1 The Francis Crick Institute, London, England, UK; 2 UK Dementia Research Institute at King's College London, London, UK; 3 Department of Basic and Clinical Neuroscience, Institute of Psychiatry, Psychology and Neuroscience, King's College London, London, UK; 4 Department of Neuromuscular Diseases, UCL Queen Square Institute of Neurology, UCL, London, UK

**Keywords:** ribo-seq, Nextflow, ribosome profiling

## Abstract

Ribosome profiling is a powerful technique to study translation at a transcriptome-wide level. However, ensuring good data quality is paramount for accurate interpretation, as is ensuring that the analyses are reproducible. We introduce a new Nextflow DSL2 pipeline, riboseq-flow, designed for processing and comprehensive quality control of ribosome profiling experiments. Riboseq-flow is user-friendly, versatile and upholds high standards in reproducibility, scalability, portability, version control and continuous integration. It enables users to efficiently analyse multiple samples in parallel and helps them evaluate the quality and utility of their data based on the detailed metrics and visualisations that are automatically generated. Riboseq-flow is available at https://github.com/iraiosub/riboseq-flow.

## Plain Language Summary

Ribosome profiling is a cutting-edge method that provides a detailed view of protein synthesis across the entire set of RNA molecules within cells. To ensure the reliability of such studies, high-quality data and the ability to replicate analyses are crucial. To address this, we present riboseq-flow, a new tool built with Nextflow DSL2, tailored for analysing data from ribosome profiling experiments. This pipeline stands out for its ease of use, flexibility, and commitment to high reproducibility standards. It's designed to handle multiple samples simultaneously, ensuring efficient analysis for large-scale studies. Moreover, riboseq-flow automatically generates detailed reports and visual representations to assess the data quality, enhancing researchers' understanding of their experiments and guiding future decisions. This valuable resource is freely accessible at https://github.com/iraiosub/riboseq-flow.

## Introduction

Translational control is an essential step in regulating gene expression. Ribosome profiling (ribo-seq) enables the investigation of the global translational landscape of cells by capturing and sequencing ribosome-protected mRNA fragments ^1– 3^. Beyond studies of translational regulation, the method has also been valuable for discovery of new translated open reading frames (ORFs) in a wide range of biological processes and organisms.

Ribo-seq is based on the ability of ribosomes to protect ~30 nt of mRNA from RNase degradation. Isolating and sequencing of these ribosome-protected fragments (RPFs, also known as ribosome footprints) offers a snapshot of the position of the ribosomes at a given time ^1^. RPFs can be used to infer translational efficiencies, their positions can reveal the identity and characteristics (e.g. frame) of the translation products, while their distribution can shed light on translational control mechanisms, such as translational pauses, ribosome residence time and upstream open reading frames ^3, 4^. Recent advances in this technique allow for the targeted study of ribosome translation based on their cellular localisation ^5^ or interaction partners, and improved experimental protocols are progressively being developed ^6^.

The wide adoption of ribo-seq has led to development of numerous computational tools and software to process the sequencing data, and for downstream applications ^7– 10^. Before extracting biological insight from ribo-seq experiments, one must ensure that genuine RPFs are captured and the data is of high enough quality, for which dedicated tools have been developed. Web-based servers and pipelines aiming to automate ribo-seq analysis are also available ^11– 17^. Workflow languages such as Nextflow ^18^ can help build, test and execute analysis pipelines consisting of a series of steps, and their integration with containerisation tools have been used to meet scalability, reproducibility and portability requirements for a number of standard bioinformatics analyses like RNA-seq ( https://nf-co.re/rnaseq) and CLIP-seq ^19^. The nf-core community has been very effective in advocating standardisation and adoption best-practices ^20^, and the standards nf-core established are valuable for advancing the development of ribo-seq data analysis pipelines.

**Figure 1.**
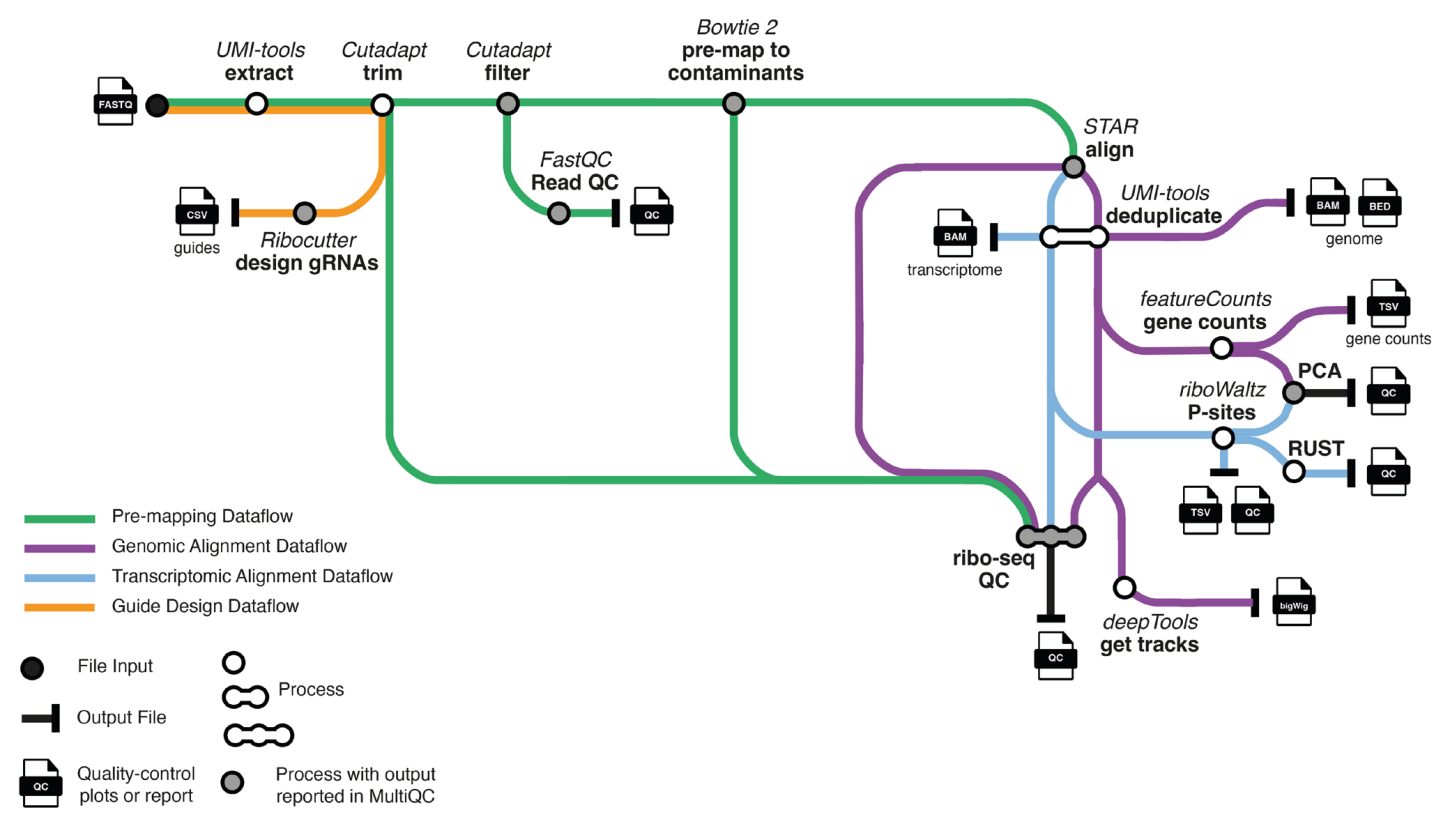
Overview of the main data processing steps of riboseq-flow. The outputs useful for most users are shown. Icons from https://nf-co.re/eager under a CC-BY 4.0 license.

Here we introduce riboseq-flow, a robust, customisable and streamlined Nextflow ^18^ DSL2 pipeline for the processing and quality control assessment of ribo-seq data ( Figure 1). Riboseq-flow simplifies the analysis process, promotes best practices, and aligns with FAIR standards ^21^, making these practices highly accessible to end-users. Riboseq-flow complements existing ribo-seq workflows ^7^, including riboviz 2 ^11, 12^ and RiboFlow ^15^ built using the Nextflow DSL1 syntax, and RiboDoc ^14^ that is Snakemake-based. While these pipelines can be used with container technology to ensure reproducibility, they differ in their ease of installation and use, customization capabilities, version control, integration standards employed to support development and collaboration, and quality control and reporting functionalities ( Table 1).

**Table 1.** Comparison of ribo-seq data analysis pipelines that integrate or can be used with container technology.

Pipeline	Implementation			Usage		Data processing			Quality control and reporting
	Workflow Language	Version Control	Continuous Integration	Installation/ Containers technology intergration	Execution	Read Trimming	UMI support	Alignment	
riboviz 2 ^11, 12^	Nextflow DSL1 *	Yes	No; integration tests available for manual run	Requires knowledge of containerization software; Complex installation.	YAML file to input all parameters (assisted by a GUI tool); Example configurations provided; CLI	3' adaptor only	Yes	Not customisable using the CLI tool	Per-sample HTML report; R/Shiny app for multi-sample analysis outside the pipeline.
RiboFlow ^15^	Nextflow DSL1 *	Not since initial release in 2019 (v0.0.1)	No	Seamless integration of container technology; Easy installation.	YAML for parameter input; example configuration included.	Customisable	Yes (not in the current release)	Customisable	External visualization required using .ribo files and same ecosystem tools ^10, 15^ ; not automated by the pipeline.
RiboDoc ^14^	Snakemake	Yes	No	Requires users to operate containerisation software and set-up a precise architecture.	YAML for parameter input; example configuration included; Reference files must be prepared in advance	3' adaptor only	No	Not customisable	riboWaltz ^22^ or TRiP diagnostics
**riboseq-** **flow** (this study)	Nextflow DSL2	Yes	Yes	Seamless integration of container technology; Easy installation.	CLI and YAML options for parameter input; parameter details in documentation; defaults provided.	Customisable	Yes	Customisable	MultiQC summary, individual PDF reports, read-length statistics, read-fate tracking, riboWaltz ^22^ P-site diagnostics, and RUST analysis ^23^ included.

Notes: **Implementation:** *DSL1 support removed by Nextflow developers with the adoption of the improved DSL2 version, so these pipelines may become outdated. Version Control enhances reproducibility. Continuous Integration makes development more robust and maintainance and collaboration easier. **Usage:** Need to edit strictly formatted YAML configuration files containing all the parameters taken by the pipeline reduces ease of use. Need to operate containerisation software directly reduces accessibility to reproducible analysis and ease of use. Need to prepare folder architecture and reference files in advance reduces ease of use and increases user workload. **Data processing:** Limited read pre-processing options reduce pipeline adaptability (e.g. to diverse library designs). **Quality control and reporting:** De-coupling QC visualisation from the main pipeline analysis steps may cause traceability and reproducibility issues. Legend: **Standard** High Medium Low

Riboseq-flow is an end-to-end pipeline that effectively addresses the gaps of existing tools by offering ease of installation and use, flexibility, and extensive quality control with specialised and general QC reports, while ensuring consistent results across compute infrastructures ( Table 1). First, riboseq-flow manages container runtime interactions, freeing users from the complexities of containerisation to maintain reproducibility. Second, riboseq-flow simplifies execution by providing a well-documented list of parameters that can be passed as command-line arguments. Pre-set sensible values are available for ease of use, eliminating the need for users to input them manually, while users desiring more control still have the option to adjust any parameter to suit their specific requirements. The pipeline's adaptability extends to different library preparation methods and organisms, and allows it to efficiently analyse many samples simultaneously, facilitating meta-analyses and comparative studies.

Importantly, riboseq-flow automates the generation of detailed, per-sample QC reports with ribo-seq specific metrics and read statistics, alongside a MultiQC ^24^ summary report. It expands beyond the typically reported ribo-seq metrics by automatically producing diagnostics such as RUST analysis ^23^ and detailed read-length specific statistics, not offered by the other pipelines. It also introduces a novel visualisation that monitors read fate through the main pipeline steps.

Built with DSL2 and a modular architecture, riboseq-flow is easily maintainable and integrates well into larger workflows. It is particularly accessible for nf-core users ^20^, and contributor-friendly due to its continuous integration (CI) framework and contributing guidelines in the documentation.

## Methods

### Implementation

Our implementation is written in Nextflow ^18^ DSL2, an evolution of the Nextflow language that makes it possible to scale and modularise pipelines. We adopted practices from nf-core ^20^ and leveraged nf-core tooling to build our pipeline. As such, riboseq-flow ^25^ is structured with modular components, each encapsulating its functionalities and dependencies, facilitated by containerisation technologies ^26, 27^. This design is combined with a configuration-driven approach that allows behaviour to be easily modified based on the target computing environment and user needs. Where possible, we incorporated modules contributed by the nf-core community ( https://nf-co.re/modules), which have the advantage of being rigorously standardised and tested.

The riboseq-flow design is depicted in Figure 1. Riboseq-flow features a sub-workflow for preparing reference files and primary ribo-seq data processing steps, structured into modules and sub-workflows.

The data processing steps automate requirements for comprehensive ribo-seq data analysis, including: read trimming and filtering, quality assessment of the sequencing reads (for example, average GC content, base composition and overrepresented sequences), pre-mapping to abundant non-coding RNA sequences to remove contaminants frequently occurring in ribo-seq data, genome alignment, UMI-based collapsing of PCR duplicates, gene-level quantification, generation of coverage tracks for genome browser visualisation, P-site identification, and comprehensive read logging and specialised ribo-seq QC. The pipeline employs standard tools and custom scripts, for which all necessary dependencies are stored in containers defined within each module.

The workflow is highly customisable. Most pipeline steps employ parameters that can be specified by the user, with options for skipping certain steps (e.g. unique molecular identifier (UMI) transfer to the FASTQ header or P-site identification), selecting preferences (e.g. read length range of interest for QC visualisations or coverage tracks format) or providing tool-specific arguments (e.g. for pre-mapping and mapping). The QC metrics, either general or ribo-seq specific, are collected during pipeline execution.

Riboseq-flow leverages continuous integration (CI) enabled by GitHub Actions, making its development and maintenance more robust. The CI workflow automatically runs riboseq-flow on a test dataset whenever a pull request is submitted for merging code branches with bug fixes or new features into the main and development (dev) branches.

### Operation

The Nextflow DSL2 implementation and containerisation of the pipeline ensure it is portable, reproducible and scalable. Riboseq-flow is easy to install and use. The only software requirements to be able to run the pipeline are Nextflow ^18^ (version 21.10.3 or later) and a container platform, either Docker ^26^ or Singularity ^27^ (version 3.6.4 or later). Nextflow handles container image retrieval and the seamless mounting of input and output files between the host system and the containers, minimising the user's interaction with container runtimes. The pipeline is compatible with personal computers, high-performance computing (HPC) systems, or cloud environments. However, for processing large genomes and utilising parallel processing for batch sample analysis, an HPC environment is advised. Users can provide a configuration file with their system’s specification, or supply their institutional profile from nf-core.

Riboseq-flow provides clear and navigable documentation, explaining run instructions, parameters and output files in detail. The pipeline includes test data and a test configuration. When the test is run (using the test profile that specifies URLs for test data and all required parameters; see Use cases section, Step 3), the pipeline is executed on a minimal dataset. This easy-to-run test helps users quickly confirm that Nextflow is set up correctly and the workflow works on their system. To run the pipeline on their own data, the user only needs to prepare a run script and a text file with input data location (see Use cases section, Step 1 and Underlying and Extended data for examples ^28, 29^).

Next, we describe pipeline operation in relation to the pipeline steps ( Figure 1). The starting point for the pipeline are the raw FASTQ files for each sample in the dataset. Users supply these using a simple sample sheet in comma-separated (csv) format, with a sample id and the path to its corresponding FASTQ file, using the --input argument. Organism-specific reference files required are a genome sequence FASTA file ( --fasta), and an annotation GTF file ( --gtf). A FASTA file containing the sequences of abundant contaminants (typically at least rRNA sequences, but tRNA, snoRNA, mitochondrial or any other FASTA sequence to be discarded in the analysis can also be added) in ribo-seq experiments should be provided when pre-mapping is enabled ( --contaminants_fasta). The annotation files are first used to generate alignment indices for pre-mapping with Bowtie2 ^30^ and genome alignment with STAR ^31^. The GTF file is also used to select one representative transcript per gene, which will be used for QC and P-site identification. The longest CDS transcript is selected by default, but if users prefer instead to provide their own transcripts list, they may do so using --transcript_info and --transcript_fasta providing the path to files formatted according to the instructions in the documentation. Moreover, users also have the option of supplying a pre-generated STAR index with --star_index. For human and mouse, a simpler alternative method to provide annotations is by specifying the --org argument, which downloads the reference files corresponding to the indicated species.

The primary ribo-seq data processing workflow proceeds with optional UMI extraction for later deduplication using UMI-tools ^32^, and 3’ and/or 5’ adapter and quality trimming using Cutadapt ^33^. If needed, a fixed number of bases can optionally be removed from either the 5’ or 3’ ends of reads, by passing --cut_end. Thus, the workflow provides trimming options that can accommodate read processing for multiple ribo-seq library design strategies. Trimmed reads shorter than 20 nt are then discarded (this threshold can be modified by the user with --minimum_length). The quality of the remaining pre-processed reads is assessed with FastQC ^34^. Pre-processed reads are subsequently pre-mapped to the contaminants FASTA using Bowtie2 ^30^. This step can be skipped using --skip_premap. The reads that do not map to contaminants are aligned to the genome using STAR ^31^. Uniquely mapped reads are kept, their transcriptomic coordinates are calculated, and PCR duplicates are optionally collapsed based on UMIs and read coordinates using UMI-tools ^32^. Next, reads counts are summarised at gene-level using featureCounts ^35^, for which the user must supply the strandedness of their library with --strandedness. These tables can be used downstream for differential expression analysis. The pipeline then generates coverage tracks using deepTools ^36^ in either bigWig or bedGraph format (chosen by the user with --track_format) with a resolution specified with --bin_width. These files facilitate exploration and visualisation in genome browsers. The pipeline then optionally identifies P-sites and generates P-site diagnostic plots ( Figure 4) and P-site counts using riboWaltz ^22^. Relevant options for this step are --periodicity_threshold; only read lengths satisfying this periodicity threshold (i.e. the percentage of read extremities in one of the three reading frames along the CDS), and --expected_length which filters RPFs based on a length range before offset calculation. While numerous P-site identification methods are available, we implemented riboWaltz ^22^ because it is a top performer in benchmarking studies ^22, 37^, is widely used by the community and delivers comprehensive diagnostics.

**Figure 2.**
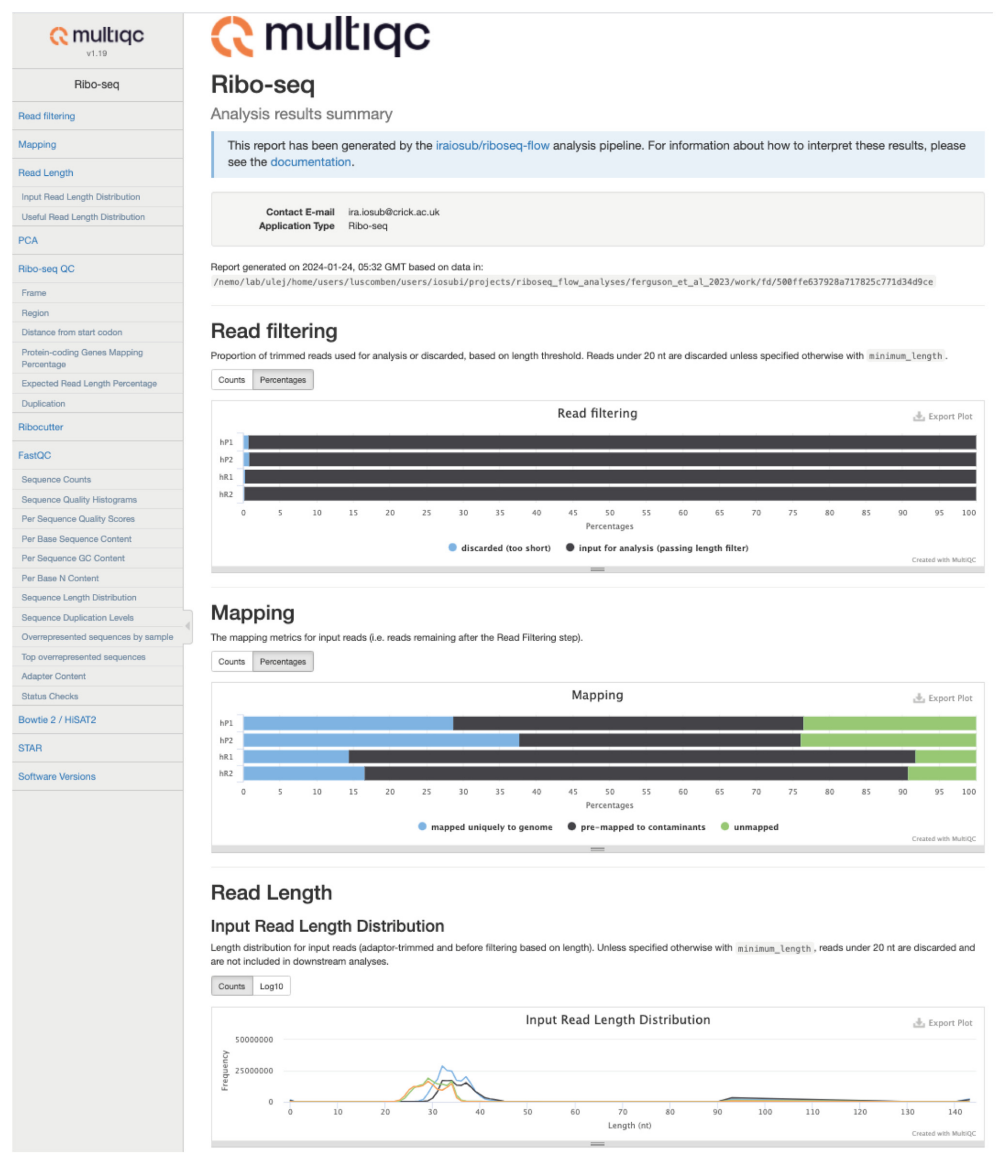
Snapshot of the MultiQC ^24^ report generated by riboseq-flow. The left sidebar helps users navigate through the report sections. A full example report for the Use case I dataset ^6^ can be browsed here. More reports for the Use case II examples ^42^ can be downloaded here for human brain data and here for mouse brain data.

**Figure 3.**
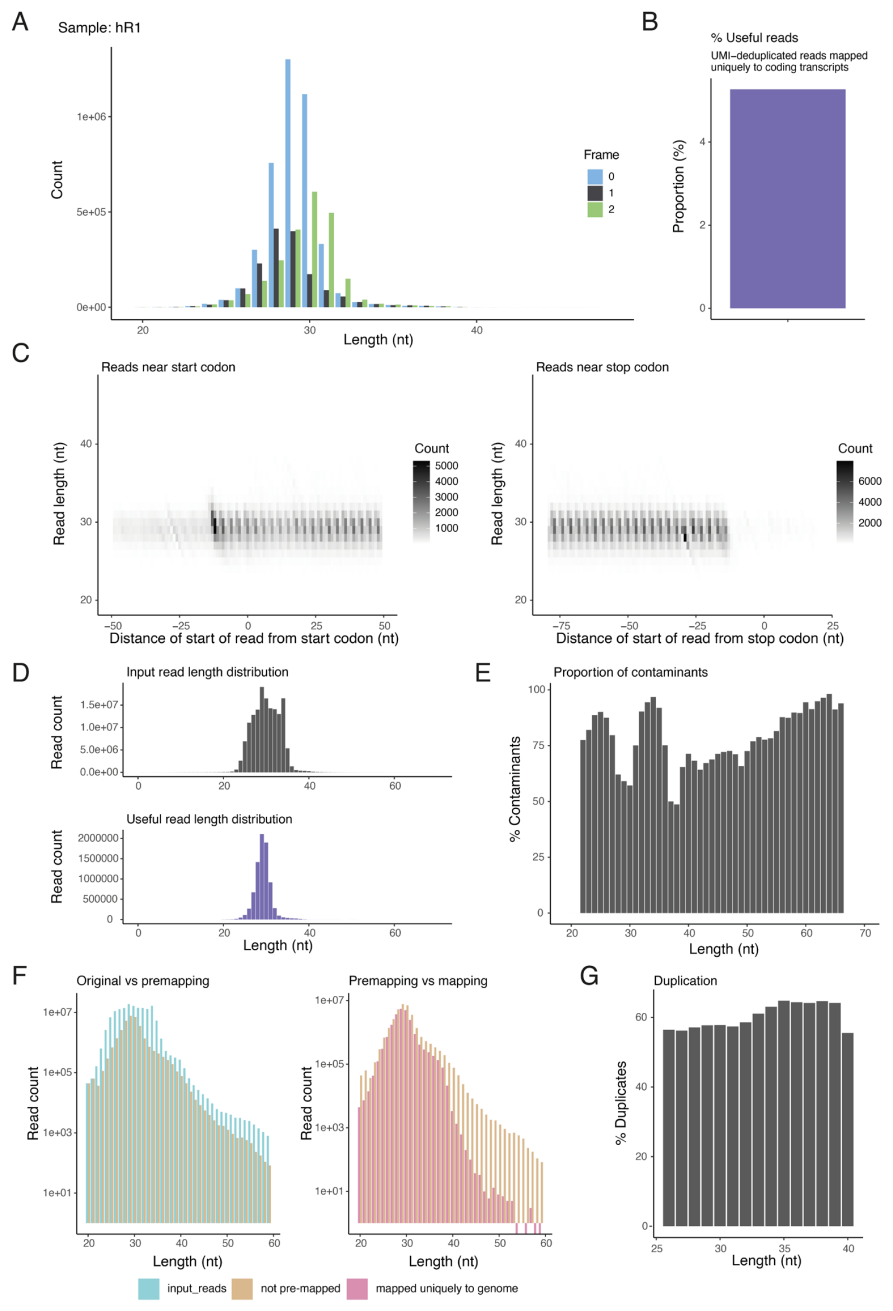
Ribo-seq QC plots with read-length stratified metrics for one RNase I 293T sample ^6^ re-analysed with riboseq-flow. ( **A**) Triplet periodicity plot showing the distribution of sub-codon positions of 5’ ends of RPFs to the protein coding regions. ( **B**) Percentage of useful reads: ratio of unique reads that mapped uniquely to protein-coding transcripts to the total number of adaptor-trimmed input reads. ( **C**) Heatmaps showing the count and position of 5’ ends of reads around the start (left) and stop codons of transcripts (right). For ribo-seq, it is expected to see the reduction of signal after the stop codon, an accumulation of read starts upstream of the start codon, and a repeating pattern every 3 nt. ( **D**) Read length distribution of adaptor-trimmed input reads (top) and useful reads (mapped uniquely to protein-coding transcripts). ( **E**) Percentage of reads mapping to abundant non-coding RNA contaminants such as rRNA. ( **F**) Number of reads of specific lengths before and after pre-mapping and genome alignment (before deduplication). ( **G**) Duplication percentage for reads of expected length.

One of the key features of the pipeline is its extensive logging and quality control. The pipeline tracks reads through every executed step of the pipeline, and uses logs and summary files generated by the pipeline software and scripts to generate three types of quality assessment outputs: (i) an interactive MultiQC ^24^ report with ribo-seq specific metrics summarised, in addition general read and alignment stats ( Figure 2), (ii) a PDF report for each sample with read-length resolved ribo-seq-specific QC visualisations ( Figure 3), (iii) interactive Sankey network diagrams showing read fate through the pipeline for each sample ( Figure 5) and (iv) diagnostic plots based on the inferred P-sites. The quality control execution can be skipped with --skip_riboseq_qc. We also recommend users to specify the expected RPF length range to a different value if the default of 26-32 nt inclusive ( --expected_length 26:32) is not fit for their experiment - this parameter is used for QC visualisation and as a read length filter for P-site identification. Finally, for users who wish to design guide RNAs for the Cas9-mediated depletion of abundant contaminants from libraries based on their data, there is an optional step that runs the Ribocutter script ^38^ on the adaptor-trimmed FASTQ file.

**Figure 4.**
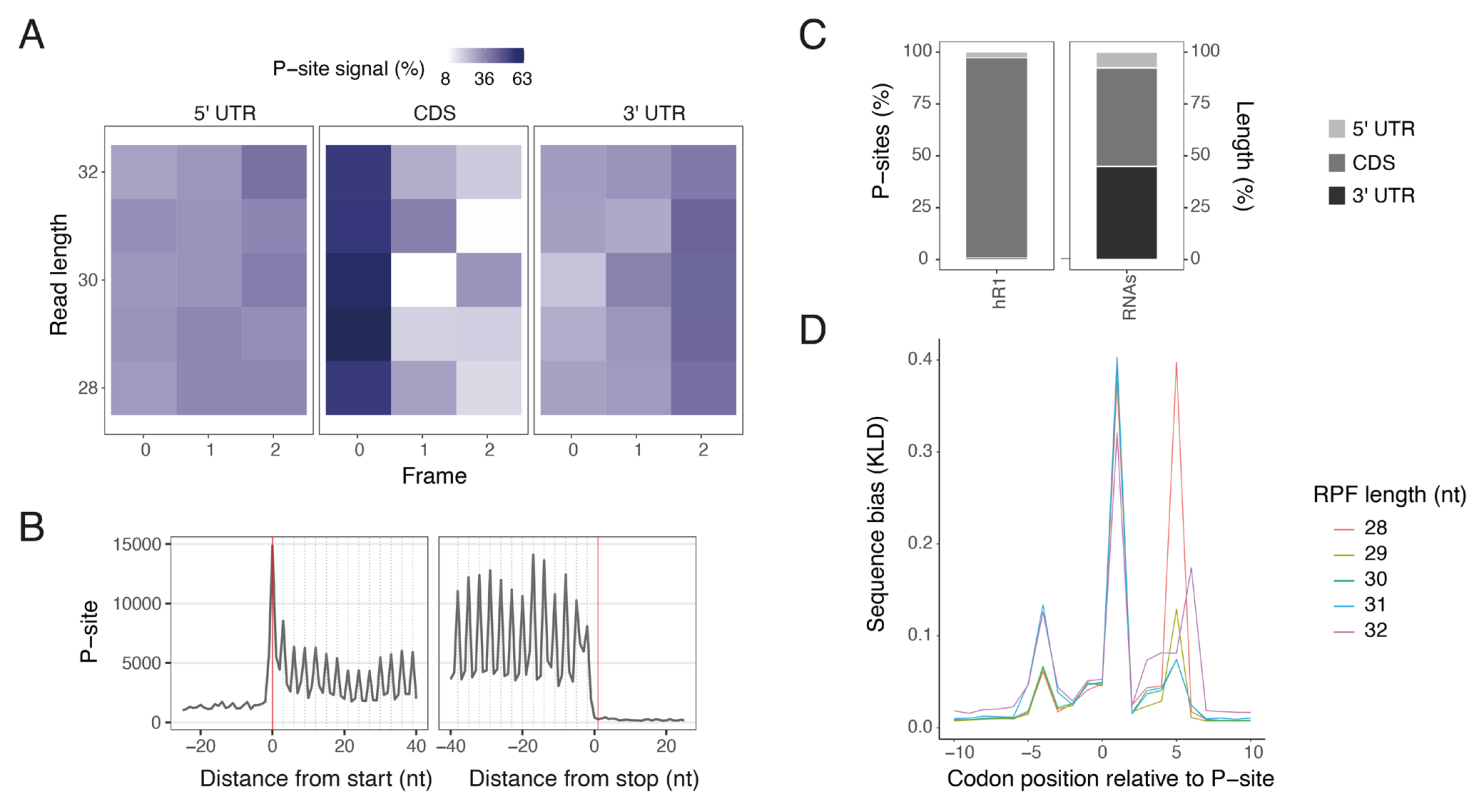
Ribo-seq QC plots generated by riboseq-flow using the riboWaltz-identified P-sites ^22^ for one RNase I 293T replicate ^6^. ( **A**) Heatmaps of P-site percentage in the three frames across untranslated and coding regions generated by riboWaltz ^22^. ( **B**) Meta-profiles showing the periodicity of ribosomes around start and stop codons generated by riboWaltz ^22^. ( **C**) P-site percentage across protein-coding transcript regions, compared to a theoretical distribution derived from feature lengths (RNAs). ( **D**) Ribo-seq Unit Step Transformation (RUST) analysis showing read-length resolved meta-profiles of Kullback–Leibler divergence as a measure of sequence bias at positions relative to the inferred P-sites (sub-sampled to 1 million reads).

**Figure 5.**
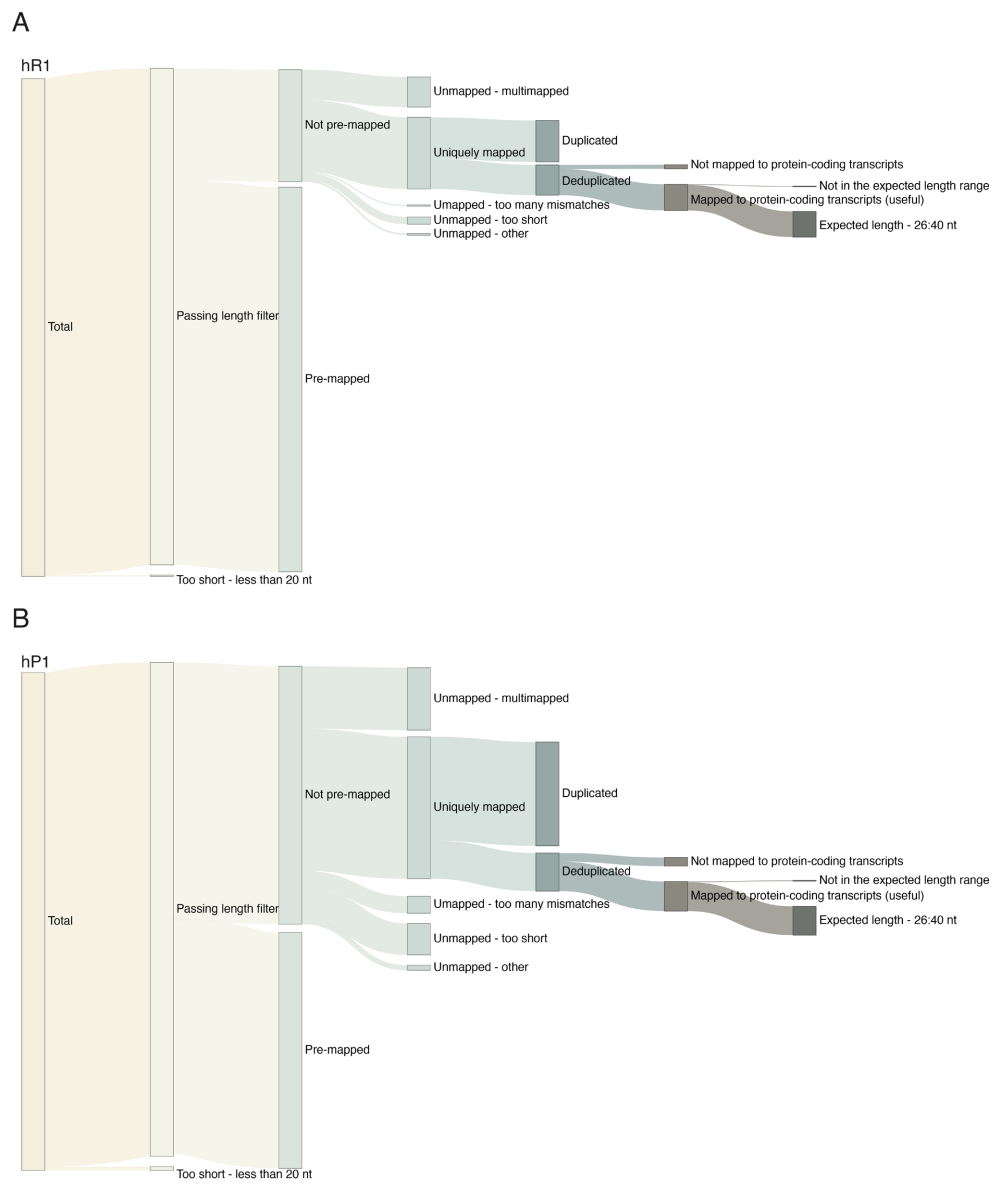
Example visualisation of read breakdown through the main steps of riboseq-flow. ( **A**) Sankey network for RNase I (hR1) re-analysed 293T ribo-seq data from 6. ( **B**) Sankey network for P1 RNase (hP1) re-analysed 293T ribo-seq data from 6.

The workflow offers extensive customization options with pre-configured default settings for ease of use, allowing users to modify these settings via CLI arguments before execution. Thus, it is user-friendly and accessible to researchers with varying levels of computational expertise. It generates output in standardised formats, compatible with specialised ribo-seq tools and suitable for visualisation in genome browsers. Uniform processing is possible for samples generated by various library preparation strategies, including ligation based, template-switching ^39, 40^ (employed by some small RNA sequencing kits such as SMARTer smRNA-Seq Kit) and OTTR ^6, 41^.

## Use cases

Below we present two use cases on full-sized public ribo-seq datasets in human and mouse, prepared using two different library strategies ^6, 42^. For both, we provide instructions on how to run riboseq-flow and describe the settings used and explain why they are appropriate for the data. We share all the results produced by riboseq-flow for Use case I (Underlying data ^28^), and Use case II (Extended data ^29^). For Use case I we describe in more depth the outputs, focusing on read statistics and the ribo-seq QC reports.

### Use case I: OTTR ribo-seq data from human cell lysates

This section includes a worked example where we processed data from a recent study describing the OTTR ribo-seq protocol ^6^. We will illustrate the steps to execute the pipeline and describe key outputs with an emphasis on QC for four human 293T cell lysate ribo-seq samples where ribosome footprints were produced with RNase I or RNase P1 as part of the study.

***Steps to process ribo-seq data using the pipeline***

Step 1: First, we download the FASTQ files from SRA (we used nf-core/fetchngs v.1.10.1) with the accession numbers from Source data. We prepare the csv sample sheet ( samplesheet.csv) as below, with sample identifiers and the location where the raw FASTQ files have been downloaded.


sample,fastq
hR1,/path/to/fastq/SRX19188681_SRR23242345.fastq.gz
hR2,/path/to/fastq/SRX19188682_SRR23242344.fastq.gz
hP1,/path/to/fastq/SRX19188679_SRR23242347.fastq.gz
hP2,/path/to/fastq/SRX19188680_SRR23242346.fastq.gz


Step 2: Download the desired version of the pipeline:


nextflow pull iraiosub/riboseq-flow -r v1.1.1


Step 3: Test the pipeline by executing it on a minimal test dataset on the command line. This step ensures riboseq-flow works on the system before running it on the data of interest.


nextflow run iraiosub/riboseq-flow -r v1.1.1 -profile test,singularity


Step 4: Run the pipeline on the command line on the data from the study, specified in samplesheet.csv:


nextflow run iraiosub/riboseq-flow -r v1.1.1 \
-profile singularity,crick \
--input samplesheet.csv \
--with_umi \
--umi_pattern NNNNNNN \
--adapter_threeprime GATCGGAAGAGCACACGTCTGAACTCCAGTCAC \
--cut_end -1 \
--strandedness forward \
--expected_length "26:40" \
--org GRCh38


This example run used our institutional profile ( -profile crick) but as mentioned in the Operation section, users should provide their own institution configuration profile. We also specified singularity as a profile because Singularity is the containerisation software on the HPC system we ran the analysis on. These are human cell-line datasets, so we provided reference genome files downloaded from GENCODE and a pre-mapping reference containing human rRNA and other contaminant sequences using the --org option. The OTTR library protocol for this dataset requires a 7 nt UMI to be moved to the read header ( --with_umi and --umi_pattern NNNNNNN) and used for deduplication. We also trim the 3’ adaptor and the last base from the 3’ end corresponding to primer +1 base (+1T or +1C in this study) ( --adapter_threeprime GATCGGAAGAGCACACGTCTGAACTCCAGTCAC and --cut_end -1). We also modify the --expected_length parameter to a wider range (26–40 nt) for QC visualisations, because RNase I yields footprints in the 28–32 nt range, whereas P1 in the 34–39 nt range ^6^.

The pipeline outputs folders (detailed in the documentation) depending on which steps are performed or skipped. The folders most users would typically find useful are: pipeline_info contains metadata and information about the pipeline execution, mapped contains BAM files with genome and transcriptome coordinates resulting from alignment to the genome, deduplicated contains the corresponding UMI-deduplicated alignments, riboseq_qc contains QC results for all samples, multiqc contains the summary QC report, coverage_tracks contains coverage track files in bigWig or bedGraph format, featurecounts contains gene-level quantification and psites contains P-site information and diagnostics. The results from this example can be browsed in full (see Underlying data ^28^), and below we focus on describing the outputs related to QC.

### Ribo-seq QC

Ribo-seq data analysis is particularly sensitive to quality issues such as read length variation, weak periodicity of the reads, and high prevalence of reads from abundant non-coding RNAs. In general, high quality libraries are expected to feature strong 3-nt periodicity and sudden, near-total reduction of aggregated read coverage immediately after the stop codons. Datasets that do not satisfy QC should be treated with caution in downstream analyses, as it is possible that many reads are derived from a non-RPF origin. Thus, QC assessment should be easily accessible to users at an early stage to provide confidence in the biological interpretation of the results. The riboseq-flow pipeline was designed to expedite going from raw data to insight, and automatically reports general sequencing and specialised ribo-seq metrics. These are presented to the end-user as a summary in an interactive HTML MultiQC report ( Figure 2) ^24^. For each sample the pipeline also outputs detailed PDF files with publication-ready plots of read-length-resolved ribo-seq QC ( Figure 3), P-site diagnostics ( Figure 4) and read-fate tracking through the pipeline steps in an interactive HTML Sankey network ( Figure 5).

In the MultiQC report, general sequencing metrics are collated from sequencing reads assessment with FastQC ^34^, read trimming and length-filtering, pre-mapping, and genome alignment. Gene-level RPF counts are used with DESeq2 ^43^ for PCA to provide insights into sample relationships, batch effects, biological variation or outlier detection. We also added additional data visualisations that inform on length-based read filtering, as well as the relative proportions of the input reads that are pre-mapped to contaminants, aligned uniquely to the genome or remained unaligned. The proportion of reads mapping uniquely to protein-coding transcripts is also reported. For these reads, we also show the regional distribution over CDS and UTRs. These metrics can inform on technical issues, degree of rRNA contamination or library artefacts. For instance, a lack of enrichment over CDS of a well-annotated organism can indicate experimental issues, or a very high rRNA content in a rRNA-depleted library can indicate inefficient depletion. Where possible, we summarised other metrics relevant for assessing ribo-seq quality: read length distribution of input reads versus those uniquely mapped to protein-coding transcripts, proportion of the library targeted with guides designed using Ribocutter ^38^, ribosome occupancy profiles based on the distribution of 5’ ends around start codons and frame distribution for the reads of expected length. The absence of features expected for high-quality ribo-seq data, such as correct footprint length and subcodon periodicity, and especially the lack of a sudden near-total decrease in ribosome occupancy after stop codons, may indicate issues with the RNase digestion and/or size selection.

While the MultiQC report gives an overview of all the samples analysed, enabling quick inspection of multiple samples at once, we strongly recommend users to carefully examine the detailed PDF report that is generated for each sample to thoroughly assess the quality of their ribo-seq data, and if P-sites were identified, the riboWaltz ^22^ diagnostic plots. Key metrics from the report for one example RNase I dataset are described in Figure 3. Reporting QC metrics stratified by length allows for a more granular assessment of data quality and helps identify potential issues or biases that may be specific to certain read lengths. In addition to conventional ribo-seq diagnostics related to features such as triplet periodicity ( Figure 3A), distributions of read 5’ ends around start and stop codons ( Figure 3C) and read length distributions ( Figure 3D), riboseq-flow provides insights into the behaviour of read populations with specific lengths during alignment and deduplication ( Figure 3 E–G). The riboWaltz QC plots are well described in the tool’s paper and documentation ^22^, and a subset of those generated by riboseq-flow are exemplified in Figure 4 A–C.

Additionally, riboseq-flow integrates transcript sequence and P-site data, applies Ribo-seq Unit Step Transformation (RUST) ^23^ for normalisation, and uses Kullback–Leibler (K–L) divergence to quantify and visualise sequence biases around the ribosome's P-site ( Figure 4D). The K–L divergence meta-profiles ( Figure 4D) highlight the overall strength of sequence biases at different positions relative to the P-site that determine detected RPF frequencies. For ribo-seq data, three peaks in a profile are typically observed: one corresponding to the codons in the decoding center, and the other two approximately located to the 5’ and 3’ ends of RPFs, reflecting the sequence-specificity of enzymes used during library construction ( Figure 4C). Ideally, the biologically-relevant peak at the decoding centre would be large in comparison to the peaks at the 5’ and 3’ ends which are due to technical biases ^23^.

Finally, riboseq-flow tracks the reads through the pipeline, and provides for each sample a clear, interactive visualisation of the read count at each pipeline step ( Figure 5). This innovative feature provides users with a complete understanding of read fate and reasons reads are filtered during execution. Based on this, researchers can make informed decisions on experimental aspects or analysis parameters. This read breakdown is particularly informative in cases where the yield of useful reads (typically reads uniquely mapped to protein-coding genes and post-deduplication) is low, by indicating at which step most reads are discarded during the workflow. This in turn can assist troubleshooting at the experimental level, and enable systematic, unbiased comparisons where variations of a protocol are performed. We illustrate this application for the RNase I ( Figure 5A) and P1 RNase ( Figure 5B) OTTR ribo-seq datasets from human 293T cell lysates ^6^. Using riboseq-flow we recapitulated the findings from the original study, that the P1 treated samples have less contaminant content (rRNA in particular) and a higher proportion of reads uniquely mapping to the genome compared to the RNase I treated lysates.

### Use case II: ribo-seq data from mouse and human brain

We provide additional worked examples of full-sized datasets analysed with riboseq-flow that the readers can refer to. We processed human and mouse brain ribo-seq data ^42^, for which the pipeline execution scripts and output files are available (Extended data ^29^).

***Steps to process ribo-seq data using the pipeline.*** Below we describe the steps to analyse the mouse brain samples with riboseq-flow, explaining the command-line arguments not already described in the OTTR ribo-seq example (Use case I).

Step 1: After downloading FASTQ files from ArrayExpress (we used nf-core/fetchngs v.1.10.1) with the accession codes from Source data, we prepare the csv sample sheet ( samplesheet_mouse_brain.csv) as below, with sample identifiers and the location of the raw FASTQ files.


sample,fastq
mouse_brain_ribo_1,ERX2819213_ERR2812382.fastq.gz
mouse_brain_ribo_2,ERX2819214_ERR2812383.fastq.gz
mouse_brain_ribo_3,ERX2819215_ERR2812384.fastq.gz


Step 2: Download the desired version of the pipeline:


nextflow pull iraiosub/riboseq-flow -r v1.1.1


Step 3: Test the pipeline:


nextflow run iraiosub/riboseq-flow -r v1.1.1 -profile test,singularity


Step 4: Run the pipeline on the command line on the data from the study, specified in samplesheet_mouse_brain.csv:


nextflow run iraiosub/riboseq-flow -r v1.1.1 \
-profile singularity,crick \
--input samplesheet_mouse_brain.csv \
--skip_umi_extract \
--adapter_threeprime AGATCGGAAGAGCACACGTCTGAACTCCAGTCAC \
--minimum_quality 20 \
--strandedness forward \
--expected_length "26:34" \
--org GRCm39 \
--outdir results_mouse_brain


The --skip_umi_extract argument was passed and --with_umi was not provided because the reads do not contain UMIs. The Phred score for trimming low-quality ends from reads was changed to 20 using --minimum_quality 20 to match the recommended value in the original publication ^42^. The footprints were generated from RNase I treated lysates, so we set --expected_length to include reads between 26 and 34 nt. Finally, since these are mouse samples, we provided GRCm39 as the organism, and modified the output folder name from the default ( results) to results_mouse_brain to reflect the type of samples analysed.

## Conclusion

Processing of ribo-seq data can still be challenging despite the many available tools. Riboseq-flow is an easy-to-use pipeline, developed to make the data analysis more accessible to researchers without bioinformatics training, while also exposing analysis parameters to advanced users. It also addresses the need to simplify the adoption of high standards in ribo-seq analysis practices, such as reproducibility across different compute infrastructures, for which we drew inspiration from nf-core guidelines. Riboseq-flow stands out in the landscape of ribo-seq analysis tools for its holistic/comprehensive approach, combining robust data processing at scale with detailed reporting, while adhering to best practices in reproducibility, portability, scalability, and version control. Key features of our pipeline are its ability to track and tally reads at each critical step and its bespoke ribo-seq quality reporting. This not only provides users with a transparent view of the read processing and quality, but also empowers them with a deeper understanding of the data, enabling more informed decisions for downstream analyses. The inclusion of a comprehensive MultiQC report as an output of our pipeline offers an insightful summary of the data, highlighting key metrics in a user-oriented and interpretable format.

To summarise, riboseq-flow streamlines ribo-seq data analysis, leads to more reliable and reproducible results, enhances overall user experience and aids in the rapid assessment of an experiment’s success. Riboseq-flow remains under active development, with plans to extend its functionality and add interactive visualisations. We both welcome additional contributions to riboseq-flow, and hope that our modules and workflow will be useful assets for integration with complementary pipelines and community endeavours in developing cutting-edge ribo-seq analysis pipelines.

## Ethics and consent

Ethical approval and consent were not required.

## Data Availability

Raw FASTQ files processed in the Use case I section ^6^ are available from the NCBI Short Read Archive under accession no. SRP419250. Below are the SRA accession codes, experiments and descriptions: SAMN32928155: nuclease digestion of biological replicate 1 of 293T cell lysate with RNase I (hR1 - SRX19188681). SAMN32928156: nuclease digestion of biological replicate 2 of 293T cell lysate with RNase I (hR2 - SRX19188682). SAMN32928153: Nuclease digestion of biological replicate 1 of 293T cell lysate with P1 nuclease (hP1 -SRX19188679). SAMN32928154: nuclease digestion of biological replicate 2 of 293T cell lysate with P1 nuclease (hP2 - SRX19188680). Raw FASTQ files processed for the Use case II section ^42^ are available from ArrayExpress under accession E-MTAB-7247. Below are the accession codes and assay names for the ribo-seq samples used: ERR2812346: human_brain_ribo_1 ERR2812347: human_brain_ribo_2 ERR2812348: human_brain_ribo_3 ERR2812382: mouse_brain_ribo_1 ERR2812383: mouse_brain_ribo_2 ERR2812384: mouse_brain_ribo_3 Zenodo: riboseq-flow results for OTTR ribo-seq data in human cells from Ferguson *et al.*, 2023. https://zenodo.org/doi/10.5281/zenodo.10572575 ^28^ Data are available under the terms of the Creative Commons Zero "No rights reserved" data waiver (CC0 1.0 Public domain dedication). Zenodo: riboseq-flow results for ribo-seq data in human and mouse brain from Wang *et al.*, 2020. https://zenodo.org/doi/10.5281/zenodo.10573242 ^29^ Data are available under the terms of the Creative Commons Zero "No rights reserved" data waiver (CC0 1.0 Public domain dedication). Software available from: https://github.com/iraiosub/riboseq-flow Source code available from: https://github.com/iraiosub/riboseq-flow Archived source code at time of publication: https://zenodo.org/doi/10.5281/zenodo.10372020 ^25^ Licence: MIT
