# Supplementary material for: Riboseq-flow: A streamlined, reliable pipeline for ribosome profiling data analysis and quality control
Source: Wellcome Open Res. 2024 Apr 11;9:179. [Version 1] doi: 10.12688/wellcomeopenres.21000.1 (PMC11153996; doi:10.12688/wellcomeopenres.21000.1)
Supplement: Peer review history

# Reviewer report: Reviewer response for version 1

**DOI:** 10.21956/wellcomeopenres.23235.r81661

**Published:** 2024-06-05

**Authors:** Pavel Baranov, Jack Tierney

**Affiliations:** 1 Department of Biochemistry, University College Cork, Cork, Ireland; 2 School of Biochemistry and Cell Biology, University College Cork, Cork, County Cork, Ireland

This manuscript describes riboseq-flow, a new Nextflow pipeline for processing ribosome profiling (ribo-seq) data. The authors position riboseq-flow as a comprehensive and portable solution for ribo-seq analysis that ensures reproducibility. Riboseq-flow provides simple installation and usage compared to some existing ribo-seq pipelines by leveraging Nextflow, containerization, and nf-core community practices. This could lower barriers for users without extensive bioinformatics expertise. The pipeline is well-documented with examples walking through the processing of published datasets. Adoption of version control, continuous integration and contributing guidelines promotes sustainability and reproducibility. Integration of ribo-seq specific QC reports into the MultiQC summary is also a valuable feature.

 In summary, this work describes a valuable contribution enabling robust and user-friendly ribo-seq data processing. The manuscript may benefit from a minor revision to address the points below. The authors have clearly dedicated significant effort to developing riboseq-flow as a resource for the community.

 Minor criticism:

 The pipeline execution worked as described in the documented use cases, except, the MultiQC config should be staged by passing it as a value channel rather than calling it in the module straight from $params.multiqc_config. This is based on the fact that MultiQC ran without issue when we ran it with conda but failed with Docker and Singularity. Manually staging the config file to the work directory and then running with Singularity also worked.

 The comparison to other ribo-seq pipelines in Table 1 does not include any available non-Nextflow/Snakemake workflows. Although the authors focus on containerized pipelines is justified, we wonder if comparisons to a selection of non-containerised pipelines would help emphasize the advance in this workflow.

 The notes and legends under Table 1 could be clarified to improve understanding. Currently it is unclear what some of them refer to. Also fix the colour in Alignment column (in the online html version, pdf is OK).

 Perhaps it is better to use 2023 update as a reference to the RiboGalaxy.

 The statement about only needing a run script and input file is potentially misleading, as the pipeline can also be executed via the command-line without a run script. Clarification on environment configuration requirements could be helpful.

 Substantial weight is placed on triplet periodicity for assessing ribo-seq data quality. More clarity on how the pipeline handles datasets with weaker periodicity, such as those from MNase digestion, would be valuable. The evidence supporting RiboWaltz as a top P-site identifier for such data could be expanded.

Are the conclusions about the tool and its performance adequately supported by the findings presented in the article?

Yes

Is the rationale for developing the new software tool clearly explained?

Yes

Is the description of the software tool technically sound?

Yes

Are sufficient details of the code, methods and analysis (if applicable) provided to allow replication of the software development and its use by others?

Yes

Is sufficient information provided to allow interpretation of the expected output datasets and any results generated using the tool?

Yes

Reviewer Expertise:

mRNA translation, ribosome profiling data analysis

We confirm that we have read this submission and believe that we have an appropriate level of expertise to confirm that it is of an acceptable scientific standard, however we have significant reservations, as outlined above.

# Reviewer report: Reviewer response for version 1

**DOI:** 10.21956/wellcomeopenres.23235.r81663

**Published:** 2024-05-31

**Authors:** Shintaro Iwasaki, Kotaro Tomruro

**Affiliations:** 1 RIKEN Cluster for Pioneering Research, Saitama, Japan; 2 Cluster for Pioneering Research, RIKEN, Wako, Saitama, Japan

Isoub et al. developed a useful and versatile pipeline for the quality check Ribo-Seq data. The Riboseq-flow can cover the many important points, that pre-existing pipelines may not reach well. The manuscript was well written. This reviewer has a few minor comments.

 1. One of the downstream analyses of this pipeline should be a differential expression analysis. This reviewer guessed the output file of the feature count could be used for this. A guideline, instruction, or navigation for such an analysis would be helpful for readers/users.

 2. For the introduction of SMARTer kit, the author could cite Ribo-lite paper (Xiong et al. NCB 2022, PMID: 35697785), which used a similar kit for library preparation.

 3. When poly(A) tailing and template switching are used for library preparation, researchers may often struggle with the identification of boundaries between ribosome footprints and high homopolymeric sequence (poly(A) and  G repeats). A short comment/suggestion should be helpful for readers/users.

Are the conclusions about the tool and its performance adequately supported by the findings presented in the article?

Yes

Is the rationale for developing the new software tool clearly explained?

Yes

Is the description of the software tool technically sound?

Yes

Are sufficient details of the code, methods and analysis (if applicable) provided to allow replication of the software development and its use by others?

Yes

Is sufficient information provided to allow interpretation of the expected output datasets and any results generated using the tool?

Yes

Reviewer Expertise:

RNA, Translation, Ribosome profiling, RNA binding proteins, Translation inhibitors

We confirm that we have read this submission and believe that we have an appropriate level of expertise to confirm that it is of an acceptable scientific standard.

# Reviewer report: Reviewer response for version 1

**DOI:** 10.21956/wellcomeopenres.23235.r81658

**Published:** 2024-05-24

**Authors:** Can Cenik, Dayea Park

**Affiliations:** 1 The University of Texas at Austin, Austin, Texas, USA

The Riboseq-flow is designed to evaluate the quality of ribosome profiling data. It allows users to customize various aspects of the data processing, including pre-mapping, mapping, and ribosome footprint size adjustments. While several similar methods have been developed in the past, this article highlights the differences and potential advantages of Riboseq-flow compared to these existing tools. The primary strengths of Riboseq-flow appear to be its ability to facilitate secondary analyses, all within a standardized output format.

 The authors provide a strong rationale for ribosome profile-specific data processing. Detailed examples of use cases illustrate the usage of specific parameters. The interpretations of Ribo-seq QC offer insights into the practical considerations of the ribosome profiling experiments. Additionally, the tool supports secondary analyses, incorporating previously developed RPF analysis tools. Overall, the manuscript is well-written. 

 Riboseq-flow simplifies analyses by reducing the need to install multiple tools to build a working environment. Additionally, the method considers both genomic and transcriptomic alignments, enabling diverse further analyses that deal with isoforms. This versatility makes Riboseq-flow an asset for researchers working with ribosome profiling data. 

 Major comments:

- Unfortunately, we were not able to test the software in detail. We encountered errors during the mapping step. In the specified working environment (Nextflow v23.10.1 with Java v17.0.11 and Docker version 20.10.12), the pipeline testing failed. Even after downloading the test data and running it manually, the same error message persisted. Clarification is needed regarding this step.  We would be happy to provide additional feedback if the authors are able to help us troubleshoot this issue. 

Executing error:

 ERROR ~ Error executing process > 'RIBOSEQ:PREPARE_RIBOSEQ_REFERENCE:GET_TRANSCRIPT_INFO (chr20.gencode.v29.primary_assembly.annotation.gtf)'

 Caused by:

 Process `RIBOSEQ:PREPARE_RIBOSEQ_REFERENCE:GET_TRANSCRIPT_INFO (chr20.gencode.v29.primary_assembly.annotation.gtf)` terminated with an error exit status (137)

 Minor comments:

- The use cases appear redundant, with Use Case II replicating the same workflow but applied to a different organism (mouse). Instead of presenting Use Case II, you could include sentences highlighting the available options for the --org parameter (GRCh38 or GRCm39). Additionally, the title mentions "ribo-seq data from mouse and human brain," but only utilizes mouse brain data as far as we can tell. Therefore, it is recommended to consider updating the title.
- Although the GitHub page provides explanations on how to use manual annotation without relying on simplified options, the manuscript lacks this part. Including relevant information about what is required for a manual run is recommended. This information would be particularly helpful for users analyzing data from non-human or non-mouse organisms. 

Are the conclusions about the tool and its performance adequately supported by the findings presented in the article?

Yes

Is the rationale for developing the new software tool clearly explained?

Yes

Is the description of the software tool technically sound?

Yes

Are sufficient details of the code, methods and analysis (if applicable) provided to allow replication of the software development and its use by others?

No

Is sufficient information provided to allow interpretation of the expected output datasets and any results generated using the tool?

Partly

Reviewer Expertise:

Computational Biology, Translation

We confirm that we have read this submission and believe that we have an appropriate level of expertise to confirm that it is of an acceptable scientific standard, however we have significant reservations, as outlined above.
